# Preventative behavioural interventions that reduce health inequities: a systematic review using the theoretical domains framework

**DOI:** 10.1186/s12889-025-22740-1

**Published:** 2025-05-23

**Authors:** Kalpa Pisavadia, Rhiannon Tudor Edwards, Ceryl Teleri Davies, Ashley Gould, John Parkinson

**Affiliations:** 1 Centre for Health Economics and Medicines Evaluation, Bangor University, Gwynedd, LL57 2PZ UK; 2 Wales Centre for Behaviour Change, Department of Psychology, Bangor University, Bangor, UK; 3 Public Health Wales, Behavioural Science Unit, Cardiff, UK

**Keywords:** Health promotion, Behavioural intervention, Theoretical domains framework, COM-B, Prevention, Gender inequalities, Ill housed, Low socioeconomic status, Poverty, Ethnic minorities, Economically disadvantaged

## Abstract

**Background:**

Wider determinants of health, such as the conditions in which people are born, grow, live, work and age influence health and well-being, often contributing to health inequities. The purpose of this systematic review is to identify preventative behavioural interventions that reduce health inequities or inequalities and to analyse which theoretical domains have been used in the intervention design and implementation.

**Methods:**

Behavioural interventions that reduced health inequities and inequalities were identified with use of the Theoretical Domains Framework (TDF). Interventions that were aimed at individuals within the axes of inequality and used the TDF in the design and implementation met our inclusion criteria. Periodicals indexed in EMBASE, MEDLINE, PsycINFO and Cochrane Library databases were selected to undertake this review. Grey literature was sought from UK local government organisations, as the sector with significant influence over these determinants. A minimum of two independent reviewers used standardised methods to search, screen, critically appraise and synthesise included studies.

**Results:**

This systematic review identified a total of 41 articles which includes *n* = 33 primary studies and *n* = 8 local government reports of behavioural interventions that reduce inequalities for populations within the axes of inequality. Most of the evidence demonstrated that behavioural interventions significantly improved health outcomes and contributed towards positive behavioural changes in health and well-being. A large proportion of the evidence base consist of interventions focusing on diet and exercise uptake (*n* = 15) specifically aimed at ethnic minorities and those of immigrants and refugee status.

**Conclusion:**

Most of the included studies incorporated elements from contemporary behavioural theory. Most health interventions identified in this review included a component to raise awareness and educate their target audience. However, whilst there was often an evidenced based rationale for use of a preventative behavioural intervention, specific frameworks were rarely used to align problems with solutions in a theoretically defined manner.

**PROSPERO registration:**

CRD42024553898.

## Introduction

Wider determinants of health, such as the circumstances of birth, growth, living, working, and ageing, significantly impact health and well-being throughout the life-course [1, 2]. The Marmot review [3] highlighted the connection between wider determinants of health and health inequalities, which have been exacerbated by over a decade of austerity measures. Vulnerable populations and individuals with protected characteristics exhibit a reduced health span compared to the most affluent in society [4]. Many communities with shared protected characteristics, such as race, disability, geography, and gender, experience high levels of multiple deprivation, which are often intergenerational and a precursor to unmet needs and inequities in health and social care [2, 5].

In England, there is a 19-year discrepancy in healthy life expectancy between individuals of high and low socioeconomic status [6]. In Wales, this discrepancy is 8 and 9 years for females and males, respectively [7]. Poorer people spend a greater proportion of their life in poor health than people living in less socioeconomic hardship. In Wales, the disparity in healthy life expectancy of males and females in the most and least deprived areas is 20 and 17 years, respectively [7]. Tobacco use, poor diet, problematic alcohol consumption, and physical inactivity are the primary behavioural factors that contribute to years of life lost or spent in poor health [8].

Altering these factors and designing effective preventative behaviour change interventions is key to improving health and health outcomes for the population as a whole [9, 10]. Behaviours that foster good health and health span while preventing illness are crucial for the future well-being of societies and for reducing the financial burden on public sector services. This is the objective of establishing effective life-course health opportunity architecture [2], though many conventional interventions adhere to a restricted medical model, overlooking key social determinants [11]. As such, the aim of this systematic review was to identify in the existing literature preventative behavioural interventions that were designed to reduce health inequities or inequalities. Furthermore, the review sought to categorise these behavioural interventions in a systematic and informative manner, as outlined below, in order to inform the design and role of future behaviour change approaches to reducing inequalities and inequity.

**Fig. 1:**
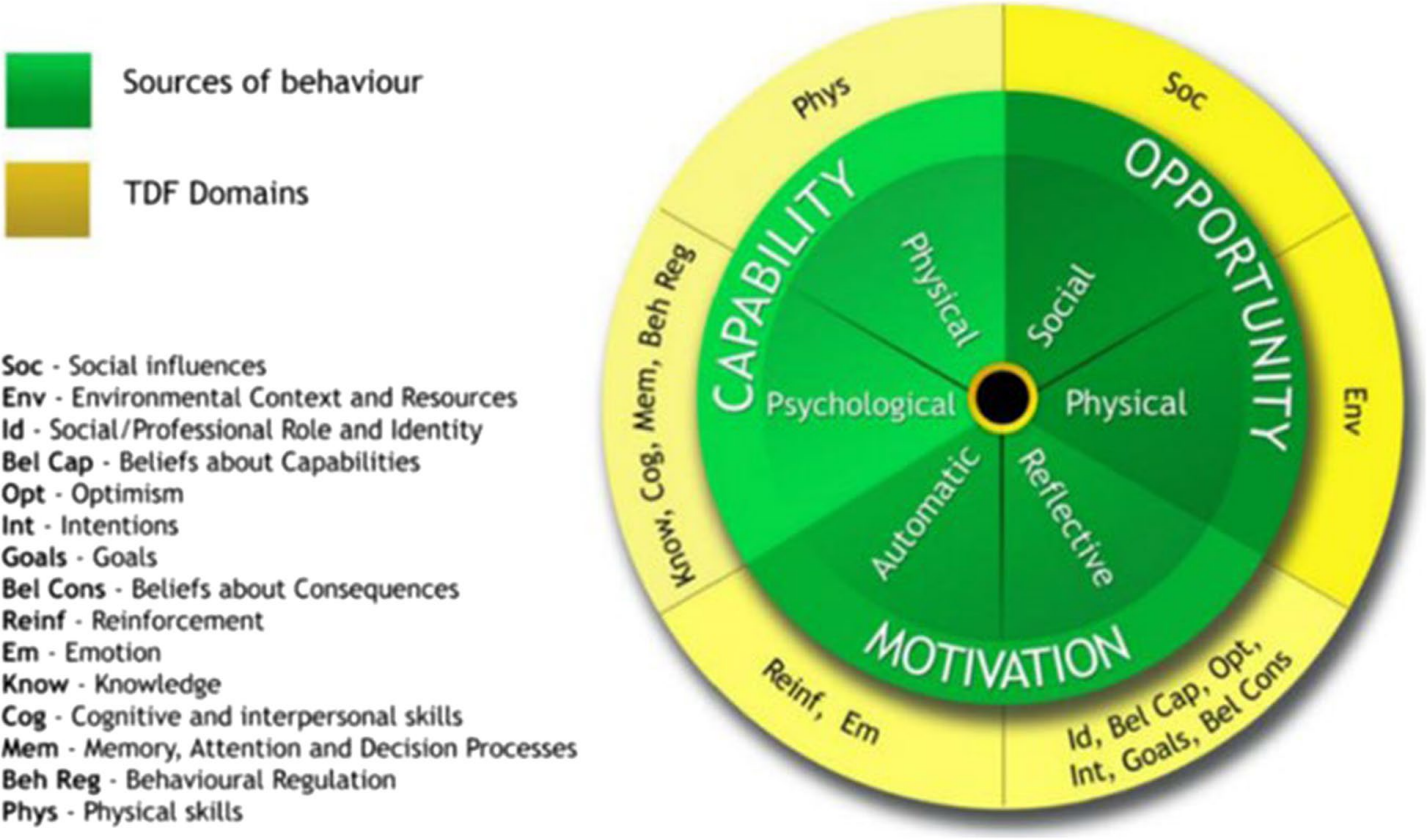
The relationship between Theoretical Domains Framework and Capability, Opportunity, Motivation = Behaviour [17]

From a theoretical perspective, an overarching consensus has gradually emerged in recent years to acknowledge at least two classes of behavioural processes, and hence to explore and understand how they function, integrate and compete [9, 12]. This dual-process approach ostensibly distinguishes between rapid, intuitive processes (type or system 1) and slower cognitive processes (type 2), with the former likely to drive behaviour in a conflicting situation [13]. Health and behavioural science researchers have developed theories of behaviour, and behaviour change, based on some mix of these multiple processes and many such theories have proliferated [14]. Whilst behaviour change is likely more effective when based on evidenced-based theory and principles [15], many applied approaches to real-world challenges are either atheoretical, employ overlapping constructs, or use different terminology for the same construct. Michie aimed to reach a consensus by developing a theoretical domains framework (TDF) to capture a definitive set of relevant constructs [16]. This approach included a ‘behaviour change wheel’ built upon the foundation of the TDF domains such that three key behavioural drivers emerge: motivation, opportunity and capability. The combination of these three drivers determines eventual behaviour, hence COM-B (Capability, Opportunity, Motivation = Behaviour) [10]. As an example, one TDF construct is Knowledge, which encapsulates the way education, information and awareness raising can influence decisions and behaviour. This construct falls primarily within the Capability element of COM-B and would predominantly engage cognitive processes from a dual-process perspective. The TDF construct of Skills encompasses both motor skills, sometimes termed habits or routines, as well as cognitive skills and the development of cognitive schema. These would fall predominantly under intuitive and automatic processes from a dual-process theory perspective. The relationship between TDF and COM-B was captured by Atkins (see Fig. 1) focusing on behaviour change and implementation challenges [17]. Researchers are increasingly adopting and validating the TDF in health and behaviour change research [17–19], and this review sought to identify its value in the context of health inequalities and inequities.

## Method

Keywords were selected to identify adult and child populations pertaining to various axes of inequality, such as health promotion, intervention strategies, prevention methods, gender disparities, inadequate housing, low socioeconomic status, poverty, and ethnic minority groups. A comprehensive search strategy was developed based on these terms and adjusted with the use of database MeSH lists. Searches were limited to articles published in English language from 2014 to 2024 and within OECD context. Periodicals indexed in EMBASE, MEDLINE, PsycINFO, and the Cochrane Library databases were chosen for this review. Grey literature was sought from UK organisations with a significant influence on wider determinants of health, such as Local Government Association, the Behavioural Science and Public Health Network, and the Behavioural Science Unit websites [20–22]. A backward citation search was conducted, scrutinising the reference lists of all included studies to ensure that no relevant literature was missed. To ensure that evidence unintentionally making use of the TDF was not overlooked, the definition of'behavioural interventions'was purposefully broad, and specific behavioural terms were omitted from the search. The eligibility criteria is presented in Table 1which incorporates an extended version of the Population, Intervention, Comparator, Outcomes (PICO) structure. Searches were completed on 25 th May 2024. The full MEDLINE search strategy is available in searchRxiv data repository [23].

**Table 1:** Eligibility criteria (population, intervention, comparator, outcomes)

	**Inclusion criteria**	**Exclusion criteria**
Participants	Adult and child populations living in areas of high deprivation, Black, Asian and minority ethnic (BAME) communities, and inclusion health groups, gender inequalities, diabetes, disabilities, prison and travellers, migration	Populations not experiencing health inequalities
Settings	Healthcare and community settings	N/A
Intervention/exposure	Interventions that reduce health inequities/inequalities and also incorporate the Theoretical Domains Framework within the intervention design and implementation	Interventions that do not reduce health inequities/inequalities Interventions that do not incorporate the Theoretical Domains Framework within the design and implementation
Comparison	N/A	N/A
Outcomes	Healthy behaviours, healthy settings, socioeconomic context, life-course health opportunity architecture	N/A
Study design	All primary studies	Opinion, commentary and editorials
Countries	Studies from the UK and countries where health services are similarly comparable to the UK, including OECD countries	Countries other than OECD countries where health services are vastly different to the UK
Language of publication	English language	Papers not available in English
Publication date	Between 2014 and 2024	Before 2014
Publication type	Published research articles Grey literature such as reports from relevant behavioural sciences websites and local governments	Pre-print

This systematic review adhered to the Preferred Reporting Items for Systematic Reviews and Meta-Analyses (PRISMA) guidelines [24]. Duplicates that were identified by Rayyan screening tool were manually removed by one reviewer. Two reviewers independently screened titles and abstracts identified through electronic searches to select potentially relevant studies. At both stages of the screening process, discrepancies were resolved through discussion between reviewers to come to an agreement on the final inclusions.

In order to provide a theoretical foundation to the analysis, an additional step was included in the screening process. The interventions within studies were critically examined by two reviewers in a second full text screening. During this process, studies were mapped in accordance with the TDF utilised in the intervention designs or TDF definitions employed within the interventions [9]. The evidence presented is framed within the ‘Behaviour Change Wheel’ (BCW), which incorporates the TDF and sits within the Capability, Opportunity, Motivation (COM-B) framework [10, 16]. The map of studies according to TDF definitions is presented in Tables 2 and 3.

**Table 2:** Map of theoretical framework for primary studies

	**Motivation**							
**Citation**	**Reinforcement**	**Emotion**	**Identity**	**Beliefs about Capabilities**	**Optimism**	**Intentions**	**Goals**	**Beliefs about consequences**
Bourke-Taylor et al., 2022 [25]			x	x				x
Bousmah et al., 2023 [26]			x	x		x	x	
Brewer et al., 2019 [27]								
Davis et al., 2014 [28]						x		
Decamp et al., 2020 [29]				x		x		
Denizard-Thompson et al., 2020 [30]								x
Dziano et al., 2021 [31]						x	x	
Eliason et al., 2017 [32]			x					
Erenoğlu and Yaman Sözbir, 2020 [33]								
Filippone et al., 2023 [34]			x	x				x
Fogel et al., 2015 [35]				x		x	x	x
Freeman et al., 2023 [36]				x		x	x	
Gilbody et al., 2019 [37]				x		x	x	x
Haas et al., 2015 [38]	x			x		x	x	
Horwitz et al., 2023 [39]								
Hughes, 2019 [40]							x	x
Kaltman et al., 2016 [41]			x			x		
Kendzor et al., 2024 [42]	x							
Knappe et al., 2024 [43]			x	x		x	x	
Kobel et al., 2016 [44]								
Lin et al., 2016 [45]	x					x		x
Lin et al., 2021 [46]			x	x		x		x
Melville et al., 2015 [47]				x		x	x	
Nagatomo et al., 2019 [48]	x							
Riley et al., 2015 [49]			x					
Ruggeri et al., 2020 [50]							x	
Soltero et al., 2019 [51]	x	x	x	x			x	x
Stormon et al., 2018 [52]								
Surratt et al., 2014 [53]			x	x	x	x		
Tanner et al., 2018 [54]	x	x	x	x			x	x
Thulstrup et al., 2021 [55]	x		x			x		
Warren and White, 2018 [56]						x		x
Wu et al., 2023 [57]						x		x

	**Opportunity**		**Capability**					**Other notable methods**	
**Citation**	**Social influences**	**Environment**	**Knowledge**	**Cognitive and interpersonal skills**	**Memory, attention and decision processes**	**Behavioural Regulation**	**Physical skills**	**User-centred**	**Systems thinking**
Bourke-Taylor et al., 2022 [25]			x	x			x		
Bousmah et al., 2023 [26]		x	x	x		x		x	
Brewer et al., 2019 [27]	x	x	x						
Davis et al., 2014 [28]	x		x	x		x			
Decamp et al., 2020 [29]			x				x		
Denizard-Thompson et al., 2020 [30]		x			x				
Dziano et al., 2021 [31]						x	x		
Eliason et al., 2017 [32]	x		x	x			x		
Erenoğlu and Yaman Sözbir, 2020 [33]			x						
Filippone et al., 2023 [34]			x	x		x			
Fogel et al., 2015 [35]	x	x	x	x		x			
Freeman et al., 2023 [36]		x	x						
Gilbody et al., 2019 [37]			x					x	
Haas et al., 2015 [38]			x						
Horwitz et al., 2023 [39]									
Hughes, 2019 [40]			x	x			x		
Kaltman et al., 2016 [41]	x		x	x	x	x	x	x	
Kendzor et al., 2024 [42]									
Knappe et al., 2024 [43]							x	x	
Kobel et al., 2016 [44]	x		x	x		x	x		
Lin et al., 2016 [45]	x	x	x						x
Lin et al., 2021 [46]			x				x		
Melville et al., 2015 [47]			x		x	x	x		
Nagatomo et al., 2019 [48]									
Riley et al., 2015 [49]	x							x	
Ruggeri et al., 2020 [50]		x							
Soltero et al., 2019 [51]			x	x		x	x		
Stormon et al., 2018 [52]		x	x						
Surratt et al., 2014 [53]			x	x		x			
Tanner et al., 2018 [54]	x		x			x		x	
Thulstrup et al., 2021 [55]		x					x	x	
Warren and White, 2018 [56]	x		x			x			
Wu et al., 2023 [57]		x	x

**Table 3:** Map of theoretical framework for local government reports

**Citation**	**Council**	**Population**	**Aim**	**Intervention**	**Category**	**Motivation**							
						**Reinforcement**	**Emotion**	**Identity**	**Beliefs about Capabilities**	**Optimism**	**Intentions**	**Goals**	**Beliefs about consequences**
Behavioural Insights Team and Cheshire East Council, 2022 [58]	Cheshire East Council	Males 40–49	To get men to think about feelings; Co-designed communication materials	Posters distributed by WhatsApp; identity focused	Well-being		x	x			x		
Alderson et al., 2020 [59]	Hartlepool Council	General population and service users	Increase early uptake of treatment services for drug and alcohol misuse	Reminders; clear expectations; MI from staff	Well-being						x	x	
The Behavioural Insights Team, 2018 [60]	Liverpool Council	General consumers	Decrease sugar consumption	Clearer sugar content labelling	Educational								
The Behavioural Insights Team, 2022 [61]	NE Lincolnshire Council	Low-socioeconomic status	Increase cancer screening rates; using reminder letters	Focusing on anticipated regret vs entering lottery	Physical health	x					x		
The Behavioural Insights Team, 2022 [62]	NE London Councils	Men aged 40–59 in deprivation deciles 1–4	Increase NHS health checks	Behaviourally informed text message; messenger and ego	Physical health		x	x					
The Behaviouralist, 2022 [63]	Sandwell Council	Unvaccinated Adults	Increase vaccine uptake; behaviourally informed messages and MI	Advocacy (peer support); addressing concerns with beliefs about consequences	Educational			x					x
The Behaviouralist et al., 2022 [64]	Wolverhampton Council	Inactive and low socio-economic residents	Increase physical activity	Variety of behaviour tools embedded in daily app messages	Physical health				x		x	x	x
Sheffield Hallam University, 2022 [65]	Yorkshire and Humber Councils	General Population	Reduce car use for short local journeys	Focus on beliefs about consequences, planning and goal setting and increase intrinsic motivation	Physical health	x			x		x	x	x

**Citation**	**Opportunity**		**Capability**					**Other notable methods**	
	**Social influences**	**Environment**	**Knowledge**	**Cognitive and interpersonal skills**	**Memory, attention and decision processes**	**Behavioural Regulation**	**Physical skills**	**User-centred**	**Systems thinking**
Behavioural Insights Team and Cheshire East Council, 2022 [58]								x	
Alderson et al., 2020 [59]		x						x	
The Behavioural Insights Team, 2018 [60]		x	x						
The Behavioural Insights Team, 2022 [61]		x							
The Behavioural Insights Team, 2022 [62]									
The Behaviouralist, 2022 [63]	x		x						
The Behaviouralist et al., 2022 [64]	x		x			x	x		
Sheffield Hallam University, 2022 [65]	x								

A single reviewer extracted data from the included papers, which was subsequently verified by two independent reviewers. Data extracted included details on the intervention aims and outcomes, the population of interest, the behavioural methodologies utilised in the design of the intervention and how it was implemented.

### Quality assessment

The Joanna Briggs Institute (JBI) critical appraisal tools were used for the quality appraisal of randomised clinical trials, cross-sectional studies, cohort studies and case reports [66–69]. However, due to the nature of this review, which specifically aims to identify the use and implementation of the Theoretical Domains Framework within the interventions, the quality of the evidence or an assessment of the certainty of evidence could not be distinguished from the JBI checklists. As the Theoretical Domains Framework is validated for its use in behaviour change and implementation research, we have essentially utilised guidance on the ‘stages of conducting TDF-based implementation research’ as a marker of quality in which to critically examine the use of theory and how well it was implemented within each intervention [9, 16, 17].

## Results

**Fig. 2:**
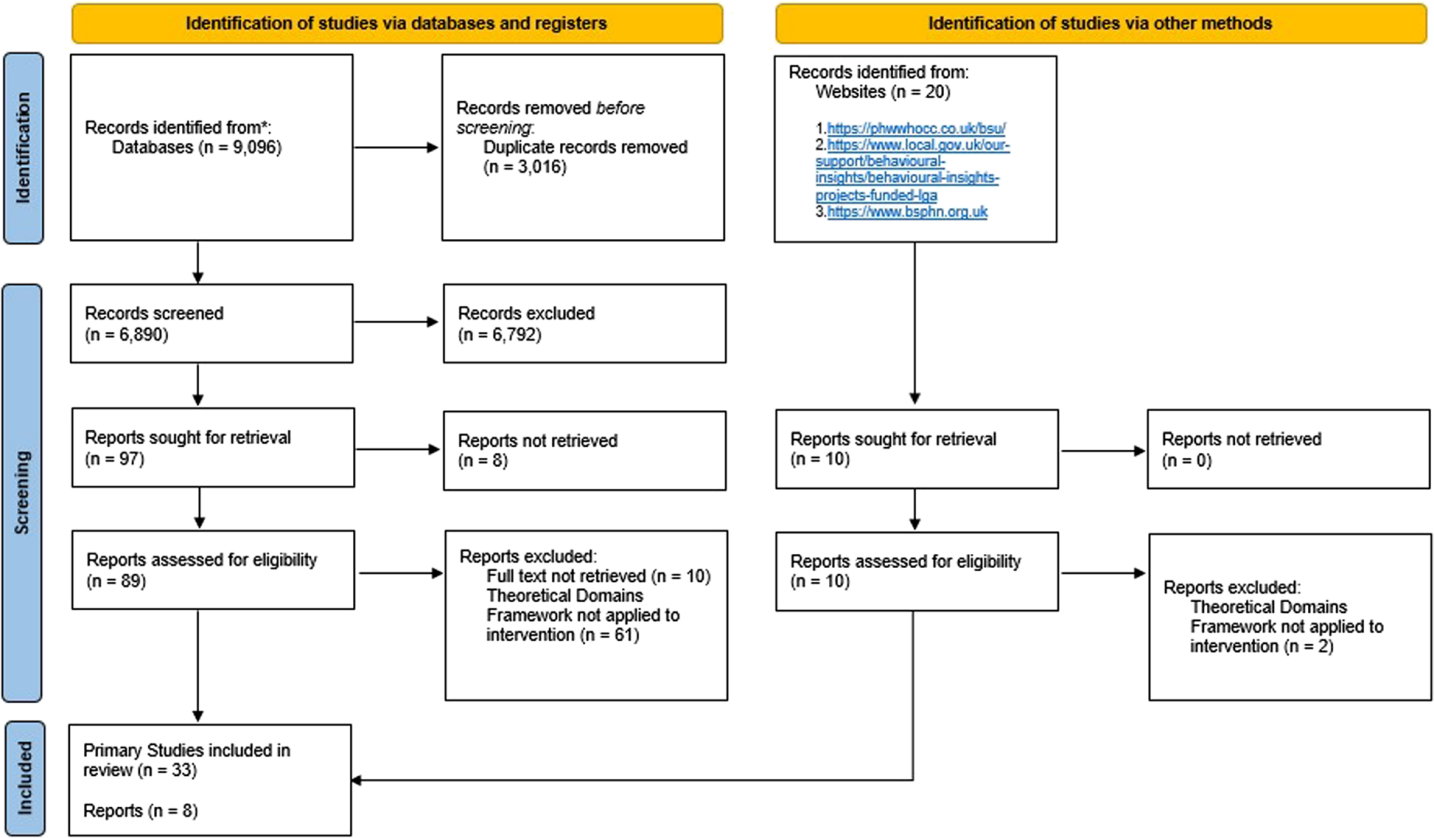
PRISMA flow diagram of included studies

After the removal of duplicates, the database searches identified 6,890 references (see Fig. 2 for the PRISMA flow diagram). Studies in which the intervention incorporated the TDF and the COM-B framework were deemed relevant in full-text screening. Following titles and abstract screening, a total of 41 articles were included in this review (primary studies, *n* = 33; local government reports, *n* = 8). These studies consisted of randomised controlled trials (*n* = 14), cross-sectional studies (*n* = 12), cohort studies (*n* = 3), case reports (*n* = 2) and non-randomised trials (*n* = 2). Additional searches of relevant websites identified local government reports (*n* = 5). All studies were assessed as moderate to high quality in accordance with the JBI checklists [66–69].

The evidence base encompassed interventions targeting adult and child populations of ethnic minorities, immigrants, and refugees. The analysis concentrated on a wide range of needs, encompassing those of women, individuals with low socio-economic status, the ill-housed and homeless, persons with physical and intellectual disabilities, older adults, rural populations, members of the lesbian, gay, bisexual, transgender (LGBTQIA) community, and individuals with mental illness. See Table 4 for study characteristics by study design, population and intervention type.

**Table 4:** Study characteristics of primary studies

Citation	Country	Study design	Population characteristics	Aim	Intervention	Category
Bourke-Taylor et al., 2022 [25]	Australia	Cohort study	Mothers (birth, adoptive, foster) of a child with a diagnosed disability	Improve mothers’ mental health, well-being and healthy behaviour	Psychoeducation and health education included a single day workshop, a workbook and access to a specially designed website with online learning package	Well-being
(Bousmah et al., 2023) [26]	France	RCT	Vulnerable, mostly undocumented immigrants in France	Empower participants in four key areas: participation, competencies and skills, self-esteem, and critical awareness (knowledge of health and social resources)	Personalised interview delivered by health mediators and social workers	Well-being
Brewer et al., 2019 [27]	Switzerland	Cross-sectional	Young Black men aged 18–29 living with and at risk for HIV	Improve socio-structural barriers to HIV services	Client-centred care coordination including pre/post release case manager, Job coordinators, LGBT-inclusive discussions on HBCU campuses, holistic health sessions	Educational
Davis et al., 2014 [28]	US	RCT	Low socioeconomic status smokers	Smoking cessation	Face to face classes,"Quit Day Retreat", guided meditation, nicotine patches	Physical health
Decamp et al., 2020 [29]	US	RCT	Latina families	Reduce health disparities	Delivered in Spanish language: interactive text messages throughout the child's first year of life, including appointment reminders, support for obtaining medicines and referrals, and illness care monitoring	Physical health
Denizard-Thompson et al., 2020 [30]	US	Cross-sectional	Individuals aged 50 to 74 scheduled to see a primary care provider and due for colorectal cancer (CRC) screening	Increase colorectal cancer screening	iPad patient decision aid called Mobile Patient Technology for Health-CRC (mPATH-CRC) for colorectal cancer screening	Physical health
Dziano et al., 2021 [31]	Australia	Cross-sectional	Rural population	Increase physical activity and healthy behaviours	1–1 motivational interviewing and follow up phone call to reinforce key messages on physical activity	Physical health
Eliason et al., 2017 [32]	US	Cross-sectional	Lesbian and bisexual women with disabilities	Increase physical activity and healthy behaviours	Five geographically dispersed interventions were grounded in nutrition education, physical activity, and group support and education	Physical health
Erenoğlu and Yaman Sözbir, 2020 [33]	Turkey	RCT	Syrian Refugee Women	Increase awareness of breast and cervical cancer	Health educational material on breast and cervical cancer	Educational
Filippone et al., 2023 [34]	US	Case study	Low-income, cis-gender Black and Latina women with history of drug use	Health literacy/economic empowerment	HIV risk reduction education, financial literacy, vocational training	Educational
Fogel et al., 2015 [35]	US	RCT	Incarcerated women	Improve sexual health behaviours/health literacy	HIV risk reduction and sexual health education	Educational
Freeman et al., 2023 [36]	US	Cross-sectional	People in subsidised housing	Economic empowerment	Goal setting, individualised action plans and motivation interviewing	Well-being
Gilbody et al., 2019 [37]	UK	RCT	People with severe mental illness	Smoking cessation	Behavioural support and Pharmacological aids, (Adaptations to the standard smoking cessation approach to better suit people with severe mental illness, including extended pre-quit sessions, cut down to quit, home visits, and coordination with primary care and mental health providers)	Physical health
Haas et al., 2015 [38]	US	RCT	Low socioeconomic status smokers	Smoking cessation	Counselling, nicotine replacement therapy (NRT) patches, personalised referrals	Physical health
Horwitz et al., 2023 [39]	US	RCT	Pregnant individuals with gestational diabetes or BMI > 25 kg/m2	Postpartum weight loss	Telephone based health coaching for postpartum weight loss	Educational
Hughes, 2019 [40]	US	Case study	Underserved Chicago population with type 2 diabetes	Improve diabetes management	Home visits, diabetes education, individual action plans, and medical referrals. Diabetes Learning Circle group course, walking group, cooking classes	Educational
Kaltman et al., 2016 [41]	US	Non-randomised trial	Trauma-exposed Latina immigrants with depression and/or posttraumatic stress disorder (PTSD)	Improve mental health outcomes	Individual component focused on reducing symptoms and increasing readiness for group sessions. Group component focused on further reducing symptoms and increasing social support	Well-being
Kendzor et al., 2024 [42]	US	RCT	Low socioeconomic status smokers	Smoking cessation	Cash and gift card incentives	Physical health
Knappe et al., 2024 [43]	Greece	RCT	Forcibly displaced Southwest Asians and Sub-Saharan Africans within a refugee camp in Greece	Improve cardiorespiratory fitness and metabolic syndrome components	Exercise and sport programme including ball sports, resistance training, martial arts, and dancing	Physical health
Kobel et al., 2016 [44]	US	RCT	First and second grade schoolchildren with a migration background	Increase physical activity and fruit and vegetable intake, decrease screen media use and soft drink consumption	Lessons on physical activity, diet, or screen media use, daily 10–15-min exercise breaks, promotion of healthy and active alternatives for children, parents'nights, regular letters to parents, and"family homework"requiring joint parent–child effort	Physical health
Lin et al., 2016 [45]	Taiwan	Cross-sectional	Ethnic minority children with asthma	Raise influenza vaccination rates	Convenient vaccine services, notifications on the availability and importance of vaccines, immunisation champion, provide education, early delivery of vaccinations for disadvantaged children	Physical health
Lin et al., 2021 [46]	China	RCT	Vietnamese immigrant mother–child pairs. Children were aged 2–6	Improve children’s dental caries; preventive maternal behaviours	1-to- 1 lessons on dental caries-related knowledge, brushing techniques, caries prevention, and information on free preventive services	Physical health
Melville et al., 2015 [47]	UK	RCT	Adults with intellectual disabilities	Increase walking and reduce sedentary behaviour	Walking advisor, educational materials, pedometers, and an individualised walking program	Physical health
Nagatomo et al., 2019 [48]	Japan	Non-randomised trial	People of low-socioeconomic status	Encourage healthy food choices	Cash-back incentive for ordering a vegetable-rich meal at participating restaurants	Well-being
Riley et al., 2015 [49]	UK	Cross-sectional	Afro-Caribbean community	Increase NHS Health Check uptake	Community-based NHS Health Checks delivered by practice staff and community health trainers	Physical health
Ruggeri et al., 2020 [50]	US	Cross-sectional	Vulnerable populations	Increase appointment attendance	Robocall and text message appointment reminder	Physical health
Soltero et al., 2019 [51]	Netherlands	Cross-sectional	Latino families (parent with an obese child between age 8 and 12)	Diabetes prevention	Nutrition education, behavioural skills training and physical activity	Physical health
Stormon et al., 2018 [52]	UK	Cross-sectional	Clients of homeless community organisations	Improve oral health care and dental appointment attendance	Dental screenings at 4 community organisations, assessment of dental treatment needs, provision of oral health education and information on treatment needs, scheduling of appointment at a dental clinic	Well-being
Surratt et al., 2014 [53]	US	RCT	Female sex workers	Reduce risk behaviours for HIV	Randomisation to either goal setting with a professional case manager or goal setting with professional case manager and recovering addict/former sex worker peer facilitator	Educational
Tanner et al., 2018 [54]	US	Cross-sectional	Individuals who identified as gay, bisexual, or transgender and living with HIV	Improve HIV-related care engagement and health outcomes	Tailored, theory-informed messages via participants preferred social media platforms (Facebook, texting, and mobile apps) to support their engagement across the HIV care continuum	Educational
Thulstrup et al., 2021 [55]	Denmark	Cross-sectional	Immigrant women	Increase exercise uptake and mental health	Participant-centred exercises focused on endurance, strength, coordination, balance, and resistance training	Well-being
Warren and White, 2018 [56]	US	Cohort study	Underserved communities	Reduce environmental tobacco smoke (ETS) exposure	Handouts, posters, visual aids, and additional background readings, training parent leaders from participating childcare centres on facilitating the"Set the Rules"workshops	Physical health
Wu et al., 2023 [57]	US	Cohort study	Patients aged 65 and older without active patient portals	Reduce disparities in COVID- 19 vaccine uptake	COVID- 19 Vaccination Telephone Outreach	Physical health

### Main findings

#### Theoretical Domains Framework (TDF)

All studies included utilised methods aligned with the TDF (*n* = 38), although only a limited number explicitly referenced the formal TDF framework (*n* = 5). The identified grey literature reports appeared more likely to base their design on the TDF (*n* = 5), suggesting either that the framework has been more readily adopted by sectors such as local governments and the third sector or that the TDF remains in the early stages of adoption as a mainstream academic structure upon which to design behavioural interventions for health-focused research. Only one primary study designed an intervention explicitly based on theory: Filippone and colleagues implemented social cognitive and asset theories in their intervention to deliver health and mental health disability services to women of low socioeconomic status [34]. All studies employed a combination of techniques in composite or stacked interventions to maximise impact. None of the studies systematically identified the most effective components.

Table 1 illustrates that a key component of most interventions was enhancing participants'knowledge and capabilities through education and awareness, alongside the development of cognitive and physical skills. A smaller portion concentrated on behavioural regulation, emphasising the management of needs and the importance of inhibitory control in decision-making. Around half of the studies recognised the importance of the physical environment (opportunity) in influencing behaviour and sought to either redesign the context to support target behaviours or to help participants understand the risks and benefits of different environments. Some studies aimed to employ normative social information to subtly influence target behaviours, including peer influence and the utilisation of role models. Some interventions focused on identity and the abilities of target individuals such as focusing on self-efficacy, beliefs about capabilities, and personal identity. Others assisted participants in goal setting, behavioural planning, interpreting intents, and overcoming obstacles. Finally, a large variety of techniques were used to increase target behaviours using motivational interventions, ranging from motivational interviewing to providing direct incentives.

#### Educational interventions

A key theme identified was the emphasis on advancing educational interventions. Ten studies were identified in this domain.

Two studies investigated diabetes support within ethnic minority populations. The STAR MAMA program targeted low-income Latina women with gestational diabetes and overweight status during pregnancy. Participants in the intervention group were provided information via an automated telephone support system and health coaching calls. The material included health themes related to postpartum care and prevention focused health themes, such as diet, exercise, breastfeeding, mood, and infant care, aimed at promoting behaviours that reduce the risk of diabetes [39]. A case study by Hughes and colleagues [40] aimed to enhance health outcomes in two Chicago neighbourhoods primarily populated by Hispanic and non-Hispanic Black residents, where diabetes prevalence was 10%, surpassing the national average of 7%, due to inadequate insurance coverage. The intervention employed a home-based strategy for managing and educating individuals regarding their lifestyle choices. A community health worker delivered diabetes education and supported individuals in formulating personalised behavioural objectives. Both studies reported improvements in health outcomes, notably a decrease in body weight among mothers in the intervention group, as noted by Hughes and colleagues.

Five educational interventions were designed to increase HIV knowledge with elements of risk reduction, health education and navigation, financial literacy and empowerment, and adopting protective behaviours. These interventions focused on a diverse population of socially and economically disadvantaged persons such as incarcerated black men [27], black and Latina women [34], incarcerated women [35], female sex workers [53] and underserved and hard to reach groups such as ethnically diverse men who have sex with men and transgender women [54]. The interventions demonstrated positive outcomes, including enhanced access to appropriate care and a reduction in missed health care appointments [27, 54]; financial empowerment, increased optimism related to employability prospects, and greater social support [34]. The study conducted by Tanner and colleagues observed a self-reported reduction in sexual partners alongside an increase in sexual health education [54].

Sandwell Council investigated the application of behavioural science in improving COVID- 19 vaccine acceptance in workplace settings. Unvaccinated individuals were provided with information presented within a behavioural framework. Individuals who received vaccinations were exposed to behaviourally framed messages aimed at promoting advocacy for increased vaccination uptake. Behavioural treatments did not significantly influence vaccination behaviour or the emergence of vaccination advocacy. The study identified significant beliefs and attitudes related to vaccinations that are associated with vaccine hesitancy. Concerns included possible side effects, beliefs about the ineffectiveness of vaccination, and scepticism about the risks associated with COVID- 19 [63].

A trial designed to influence consumer decision-making and decrease sugar intake in Liverpool implemented stop signs on high-sugar chilled drinks in two hospital stores, resulting in a 7.3% reduction in sales of high-sugar beverages. During the period when signs were displayed, there was a slight increase in sales of lower sugar alternatives, suggesting that consumers substituted high sugar beverages with these options [60].

An initiative aimed to influence behaviour change by improving health literacy among Syrian refugee women living in Turkey. A RCT was performed to evaluate the efficacy of health educational materials to improve knowledge and awareness about mammography, Pap smear tests, self-breast examinations, and HPV vaccination for breast and cervical cancer. The interventions resulted in improved health education and awareness concerning breast and cervical cancer [33].

#### Physical health interventions

This review included 21 studies that investigated the promotion of positive physical health behaviours.

Two studies included interventions which aimed to improve the physical health of ethnic minority children by communicating educational material and information to parents and providing accessible services. This included a study to improve flu vaccination rates, which also included an immunisation champion to enhance vaccination uptake [45], a lay health adviser intervention to improve dental caries [46], and a peer-to-peer parent training intervention to mitigate the effects of environmental tobacco smoke [56]. All three studies evidenced an improvement in the preventative behaviours of parents.

Two studies aimed to examine attitudes, beliefs, and behaviours related to healthcare and enhance participation in NHS Health Check programmes. One was community-based, incorporating outreach events and was designed in a user-centred approach with an Afro-Caribbean community in England. The second study was aimed at men aged 40–59 in socioeconomically disadvantaged areas of Northeast London. The intervention involved an SMS invitation for the NHS Health Check, containing a link to a voice note utilising the principle of'people like you'. Patients were identified by name; specific terminology was employed, including phrases such as"You are at the top of the queue"and"A spot has been reserved for you,"and the objectives of the Health Checks were clearly articulated [62]. NHS health checks motivate attendees to adopt healthy lifestyle changes. Additionally, these health checks enabled attendees to engage with healthcare professionals about further health issues and promoted social interaction by encouraging discussions of test results among peers [49].

Three e-health interventions were identified that employed a combination of telephone calls, emails, and text messages to promote attendance at health appointments. Two studies examined older individuals [30, 57]. An additional intervention focused on individuals facing multiple deprivation [50]. All three interventions resulted in increased appointment attendance rates relative to control groups without contact. A further e-health intervention employed the situated Information, Motivation, Behavioural Skills (sIMB) model to inform the development of an interactive text messaging service targeting Latina parents during their child's first year of life. The model enabled the creation of intervention components aimed at improving parental information, motivation, and behaviours that foster engagement with healthcare. The components were adjusted to reflect the specific characteristics influencing Latino families, considering personal, cultural, situational, and structural dimensions. The intervention group exhibited a reduction in Emergency Department (ED) utilisation and an enhancement in flu vaccine uptake [29].

A pre-post study aimed to identify key barriers to colon cancer screening uptake and to develop evidence-based strategies for targeted interventions. This included reminder letters sent by General Practitioners (GPs) to patients who did not respond to their invitation from the national screening program was supplemented with two behavioural strategies:"anticipated regret messaging"—prompting individuals to contemplate the potential emotional consequences of receiving a late diagnosis—and offering entry into a lottery as an incentive for undergoing screening. There was a significant rise in the number of patients who returned their screening kit after receiving the letter, with the highest rate observed during the 6 weeks after the letter's delivery [61].

Five studies evaluated interventions to improve physical activity, including motivational interviewing following a health age assessment within a rural Australian population [31]. An active travel initiative employed a mixture of strategies such as motivating statements, goal setting and planning in order to replace car journeys with walking or cycling [65]. An exercise and sport intervention among individuals residing in a refugee camp in Greece was designed according to self-determination theory, which highlights psychological needs such as autonomy, competence and relatedness. Motivational strategies were employed through an internal chat group and direct interactions. Participants demonstrated intrinsic motivation via autonomy, with positive feedback improving competence and facilitating opportunities for social connection, thus promoting relatedness [43]. Nudges, such as in-app push notifications, significantly enhanced all outcomes within a six-week walking program aimed at low socioeconomic and inactive populations integrated components of the COM-B model with behavioural concepts including goal setting, commitment, social comparison, and motivational feedback [64]. Long- and short-term behavioural change was evident in the majority of these studies. However, a walking program that was based on the trans-theoretical and socio-cognitive models of behaviour change and utilised motivational interviewing and goal setting to reduce sedentary behaviour among individuals with intellectual disabilities was unsuccessful. This was partly attributed to the challenges of creating a behaviour change intervention for adults with intellectual disabilities that complies with guidelines for interventions targeting disadvantaged groups [47].

Two interventions aimed at enhancing physical activity and nutritional education were implemented for children from immigrant families. Both interventions included a behavioural change or training component and employed family-based, culturally relevant strategies to promote healthier lifestyles in disadvantaged communities [44, 51]. Soltero and colleagues [51] observed a significant reduction in parental weight, stable weight in children, and an improvement in the quality of life for both parents and children. Although there was a decrease in physical activity and no notable difference in fruit and vegetable consumption in the intervention conducted by Kobel and colleagues, there was a significant reduction in screen media and soft drink consumption [44].

An overwhelming amount of evidence of behavioural interventions aimed at smoking cessation was identified. Four studies that explicitly stated the use of behavioural methods in the intervention were included. Three of these studies focused on individuals of low socioeconomic status and employed a variety of methods, including nicotine replacement therapy, telephone quit lines, community referrals, counselling, and extensively validated therapeutic modalities, which encompassed pharmacological interventions, skill training, and group support [28, 38, 42]. Gilbody and colleagues [37] conducted a study on individuals with severe mental illness, utilising modified behavioural support and pharmacological interventions specifically designed for this demographic. All four studies indicated a higher rate of smoking cessation in comparison to the intervention groups.

#### Well-being interventions

Ten studies evaluating interventions to increase mental health and well-being were identified.

Three studies targeted the mental and physical well-being of immigrant populations. This included a user-centred physiotherapist-supervised exercise programme, which delineated key characteristics, such as privacy, a community-focused environment, and the development of a trustworthy connection with physiotherapists [55]. A mental health intervention for Latina immigrant women who have experienced trauma comprised of behavioural activation (BA), and a component of Cognitive Behavioural Therapy (CBT) and motivational interviewing [41]. An empowerment intervention targeting immigrants from sub-Saharan Africa residing in precarious conditions in the Greater Paris area comprised motivational interviewing executed by health mediators and social workers. The improvement in participants'knowledge and capacity to utilise health and social services was attributed to the health empowerment approach [26].

Another empowerment intervention aimed at mothers of children with diagnosed disabilities resulted in a decrease in the prevalence of depression, anxiety, and stress. The one-day online workshop comprised six modules focused on healthy lifestyle redesign, psychoeducation, and health education regarding mental wellness, stress management, anxiety, and coping strategies [25].

A six-week well-being challenge designed to enhance men's emotional awareness and facilitate constructive changes for improved mental well-being demonstrated a significant improvement in well-being among the intervention group. The intervention utilised WhatsApp messenger for communication, whereas the control group received conventional notifications regarding the 'Well-being Challenge' [58].

A study comparing the effects of physical exercise, nutritional education, and mindfulness interventions on lesbian and bisexual women, both with and without disabilities, demonstrated comparable outcomes for both groups across the majority of measures. Lesbian and bisexual women with disabilities demonstrated more significant enhancements in physical health, quality of life and dietary intake of fruits and vegetables compared to their counterparts without disabilities despite marked differences in socio-demographic and health factors at baseline [32].

A facilitated access pathway model was developed in Australia to enhance dental care accessibility for homeless and disadvantaged adults. Participants were classified as priority populations, defined as individuals who experience significant adverse effects from inadequate oral health and encounter obstacles in accessing oral health care. Participants were evaluated by volunteer dentists, oral health therapists, and dental students in community-organised settings. Participants received instructions on maintaining oral health, detailed explanations of treatments, and a scheduled visit to a dental facility. Of the total participants (*n* = 64), 85% attended their initial appointment at the dental clinic [52].

A one-week initiative was implemented at 26 local restaurants in Japan to address socioeconomic inequality. Positive reinforcement was employed through monetary rewards for customers selecting vegetable-rich meals. During the intervention period, there was a significant increase in the number of meals ordered with high vegetable content compared to the control period [48].

A program utilising Community Health Workers (CHWs) was established in subsidised housing in New York City to meet the health needs identified by residents. CHWs interacted with individuals via goal-setting exercises, personalised action plan development, and motivational interviewing to tackle issues such as food insecurity, rental assistance, medical care, and transportation to appointments. This was accomplished by assisting residents in collecting necessary documentation, guiding them to appropriate agencies, scheduling appointments, facilitating applications for emergency rental assistance, and coordinating transportation. Following the intervention, a notable decrease was observed in the number of individuals experiencing food insecurity and challenges in meeting rent obligations [36].

A project conducted by the local government association in Hartlepool aimed to increase the uptake of services for drug and alcohol misuse by assessing the acceptability of the services and redesigning them based on the needs and behaviours of its service users. Although there was a slight increase in the number of missed appointments following the redesign, the number of individuals engaging in treatment increased by 2.2% [59].

## Discussion

The conditions in which people are born, grow, live, work and age lead to inequities in power, money and resources and underpin the social determinants of health [70]. This review aimed to identify the existing evidence of preventative behavioural interventions that reduce health inequities or inequalities and to analyse which theoretical domains had been used in the intervention design and implementation.

Since the COVID- 19 pandemic, there has been a rejuvenation in interest in the role of behavioural science in supporting improvements in health, whether through treatment and rehabilitation (e.g. behavioural adherence) or in preventative measures. Nonetheless, this review identified a mix of studies from the recent decade that utilised behavioural techniques to reduce inequity. For example, goal-setting and motivational interviewing have been shown to empower individuals to achieve self-determination [36, 41]. Several interventions equipped participants with the tools and resources to navigate the health system autonomously, minimise risk, or develop coping methods [25, 26, 34].

A specific objective of this review was to determine if studies were purposefully designed with a specific behavioural theory in mind or favoured one type of intervention approach over another. Most health interventions included a component to raise awareness and educate their target audience with a view to prevention of avoidable ill health, disability and premature death [27, 33, 35, 40, 53]. This would seem a logical and necessary first step in changing behaviour. Although the ‘nudge’ approach, popularised in the 2000 s by Thaler and Sunstein [71], advocates a more subtle and non-conscious, or implicit, approach to behaviour change, it appears that such an approach does not appear to have noticeably made it into mainstream research in this domain. Conversely, it has been adopted as an approach to influence clinical behaviours in healthcare settings [72, 73]. However, most interventions were multi-component and often combined education with other elements that could be defined as engaging the autonomic system—such as cognitive or physical training to establish new skills or habits. The majority of studies created interventions that combined techniques from all three overarching categories of the theoretical domains (COM), though none framed their intervention designs in those terms. While several studies were based on evidence-based approaches, such as mindfulness or CBT, few provided a theoretical basis for the rationale behind implementing such approaches or how they fit with other elements of the intervention [14]. This may reflect a lack of input from academia in real-world implementation studies, demonstrating a gap in ‘capability’ around behavioural science theory and practice.

Several papers identified in the initial search explicitly referenced COM-B or the theoretical domains framework; however, these primarily described intervention designs rather than fully delivered and evaluated studies. The grey literature indicates the explicit application of TDF and COM-B in intervention design, suggesting that this framework is gaining traction. It is noteworthy that the majority of studies employed interventions that integrated components from all three theoretical domains. The prevalent combinations included goal setting, motivational interviewing, and planning, alongside beliefs regarding consequences or abilities from the Motivational category; normative influences derived from peer interactions or role modelling within the Opportunities category; and education, behaviour regulation, and skill training (cognitive and physical) from the Capability category. Common behaviour change techniques involved promoting intrinsic motivation and personal autonomy, grounded in self-determination theory [74], alongside approaches to improve self-efficacy and confidence in personal abilities. Approaches categorised as opportunities involved identifying suitable contexts for facilitating change, e.g., community centres for treatment and employing peer support, including community support, to share cultural norms and role model appropriate health behaviours, e.g., vaccination uptake. Therapeutic approaches, including CBT or mindfulness, were integrated with other behaviour change interventions to specifically address mental health challenges while promoting positive change. Given that behaviour is often triggered automatically by stimuli in the environment, several studies used technological solutions to prime target behaviours, such as the use of in-app push notifications, text messages, or telephone calls [75].

A key strength of basing an intervention in theory is that the analysis of the problem can lead to insights into potential successful solutions. If a barrier to the target activity is recognised as a lack of opportunity, such as insufficient access to a community centre, the solution design might focus on improving that opportunity. None of the reviewed studies employed the COM-B model or the TDFs to align the issue with the offered solutions. None of the studies identified in this review specifically attempted this kind of process in matching the problem space with the solution space in a theoretically defined manner. Many of the interventions acknowledged the likely problem causes, i.e., numerous target groups were recognised as lacking knowledge or awareness of the desired behaviours. Although there was frequently an evidence-based rationale for the application of interventions, e.g., CBT or Mindfulness, there was a lack of consideration regarding how theoretical domains might map between problem behaviours and solutions.

## Strengths

To our knowledge, this is the first systematic review of interventions to reduce health inequities that are underpinned by behavioural science theory. This review identified a substantial evidence base comprising a variety of high-quality study designs and methods, facilitating a thorough overview of the topic. All studies employed methods consistent with the TDF, though only a few explicitly cited the formal TDF framework. The identified grey literature reports used the TDF in their design, suggesting either an increasing adoption of the framework by the third sector, including local governments, or a limited mainstream acceptance of the TDF as a foundational structure for designing behavioural interventions in health-related research. All studies used a combination of techniques; however, none systematically identified the most effective components. While evidence-based rationale for interventions like CBT or Mindfulness was often present, there was insufficient consideration of how theoretical domains could align between problematic behaviours and their respective solutions.

## Limitations

The relevant JBI Checklists were used to assess the quality of the included articles [67]. However, as the TDF is validated for its use in behaviour change and implementation research, we utilised this as a marker of quality in which to critically examine the use of theory (see Tables 2 and 3) and implementation within each intervention [9, 17]. Whilst the eligibility criteria for this review were constructed with the aims and objectives of the review in mind, due to the lack of available tools, there is a possibility that selection bias may have occurred in the additional screening stage when selecting studies that had utilised or implemented theory.

## Implications for policy and practice

Most interventions were multi-component, integrating teaching with additional elements that engaged the automatic system, including cognitive or physical exercise to develop new skills or habits. Most studies employed methods from all three overarching categories of the theoretical domains (COM-B), though none framed their intervention designs in those terms. Although numerous studies incorporated evidence-based approaches, including mindfulness and cognitive-behavioural therapy, few provided a theoretical basis for the rationale behind the implementation of these approaches or their integration with other intervention components. This may reflect a lack of academic input in real-world implementation studies, demonstrating a gap in ‘capability’ around behavioural science theory and policy design and implementation.

## Conclusion

This systematic review found substantial evidence of preventative behavioural interventions that aimed to reduce inequities among populations affected by various axes. Evidence indicates that interventions significantly enhanced health outcomes and facilitated positive behavioural changes in health and well-being. Most of the studies analysed integrated components from all three theoretical domains into interventions, with explicit use of the TDF and COM-B frameworks most evident in the grey literature. Health interventions examined in this review incorporated elements aimed at increasing awareness and educating their target audience within the sphere of preventing avoidable ill health, disability and premature death. Despite the presence of an evidence-based rationale for the use of an intervention, neither the COM-B model nor the TDF was employed to systematically align problems with solutions in a theoretically defined way. A prudent future strategy would be to increase capability and capacity in applied behavioural science in order to optimise the impact and efficacy of targeted preventative health interventions.

## Data Availability

No datasets were generated or analysed during the current study.
